# Monopolar versus bipolar transurethral resection of lateral wall-located bladder cancer under obturator nerve block: a single center prospective randomized study

**DOI:** 10.1590/S1677-5538.IBJU.2020.0568

**Published:** 2020-12-20

**Authors:** Deniz Bolat, Serkan Yarimoglu, Mehmet Erhan Aydin

**Affiliations:** 1 University of Health Sciences Bozyaka Training and Research Hospital Izmir Turkey University of Health Sciences, Bozyaka Training and Research Hospital, Izmir, Turkey

**Keywords:** Urinary Bladder Neoplasms, Transurethral Resection of Prostate, Prostatectomy

## Abstract

**Introduction:**

The aim of the present prospective-randomized study was to compare perioperative outcomes and complications of bipolar and monopolar TURBT for lateral wall-located non-muscle invasive bladder cancers (NMIBC) under obturator nerve block (ONB).

**Patients and Methods:**

80 patients who underwent TURBT for lateral wall-located primary bladder tumors under ONB from March, 2016 to November, 2019 were included in the present study. The patients were randomized equally into two groups; monopolar TUR (M-TURBT) and bipolar TUR (B-TURBT). The primary and secondary outcomes were safety (obturator jerk and bladder perforation) and efficacy (complete tumor resection and sampling of the deep muscle tissue).

**Results:**

Obturator jerk was detected in 2 patients (5%) in M-TURBT while obturator jerk was not observed during B-TURBT (p=0.494). Bladder perforation was not observed in both groups. All of the patients underwent complete tumor resection. There was no significant difference in muscle tissue sampling (67.5% vs. 72.5%, p=0.626) and thermal tissue damage rates (12.5% vs. 25%, p=0.201). The majority of complications were low-grade and the differences in Clavien grade 1-3 complications between groups were not statistically significant.

**Conclusion:**

In the treatment of lateral-wall located NMIBCs, either M-TURBT or B-TURBT can be safely and effectively performed by combining spinal anesthesia with ONB. Even so, it should be taken into consideration that low-grade postoperative hemorrhagic complications may occur in patients who undergo M-TURBT.

## INTRODUCTION

Bladder cancer is the tenth most common and the thirteenth most deadly cancer worldwide. Its incidence is rising steadily, especially in Europe and other developed countries (1). Approximately 75% of BCs do not involve the muscle wall of the bladder and are called non-muscle invasive bladder cancer (NMIBC) (2). Transurethral resection (TUR) is the gold standard for the diagnosis and initial treatment of bladder tumors (3). Maximal TUR of bladder tumors (TURBT), which is defined as macroscopically complete resection of the bladder tumor when safely possible, is critical to successful treatment in mono- and multi-modality regimens (4).

Traditionally, monopolar energy systems have been preferred to be performed in resection of bladder tumors. However, bipolar devices can also be used safely and effectively in the treatment of these tumors (5). In monopolar energy system, electric current passes through the patient’ s body and reaches a skin pad. However, bipolar system is completed locally and energy is confined at the resection site between the two poles situated on the tip. Energy is transmitted from the loop electrode into the saline solution allowing it to evaporate and form an interface layer of gas around the loop (6). In monopolar system, electrical energy is directed into the tissues, where its electrical resistance creates a high temperature. However, in bipolar systems, radiofrequency energy converts the conductive medium into a plasma field of highly ionized particles disrupting the organic molecular bonds between the tissues at a much lower temperature as which may reduce the thermal damage to the surrounding tissues (7). Furthermore, hypotonic and non-saline irrigation fluids such as 1.5% glycine and mannitol are used in monopolar energy systems. However, bipolar devices can be used with conductive fluid medium (normal saline) instead. Therefore, bipolar systems prevent patients from having TUR syndrome, which is caused by the absorption of hypotonic irrigation fluid and the resultant electrolyte imbalance (8). In addition, bipolar TURBT (B-TURBT) has some other advantages over monopolar TURBT (M-TURBT) such as a lower incidence of adductor muscle contraction resulting from adductor contraction and obturator jerk and less damage to the site of resection from the cautery procedure (9, 10).

Modern laser technology has led to new alternatives to conventional TURBT (cTURBT). The two most commonly used lasers at present are holmium (Ho:YAG) and thulium (Tm:YAG), Various research groups have already reported promising results for laser en bloc resection of bladder tumors (ERBT). However, not every hospital has access to laser devices since they cost much. Using an electrical current instead of lasers may be a promising alternative (11).

Obturator nerve originates from the lumbar plexus of L2 to L4 and both motor and sensory nerve fibers passes through the obturator foramen of the pubis to innervate the adductor muscles of the thigh. It comes to close proximity to the prostatic urethra, bladder neck, and inferolateral bladder wall (12). Garcia Rodriguez et al. reported that 46.8% of all bladder tumors are located at the lateral wall of the bladder (13). In TURBT procedure, this proximity can cause the stimulation of the obturator nerve when the bladder is distended with irrigation fluid. At this point, involuntary leg movements are caused. These involuntary leg movements increase the risk of developing complications, such as incomplete resection, bladder perforation, extravesical dissemination of the tumor, and vascular injuries (12). Prentiss et al. developed obturator nerve block (ONB) technique in 1965 in order to avoid involuntary leg movements during TURBT (14). Studies since then have shown that ONB performed with various techniques makes the TURBT procedure safer (15-18).

Despite the ONB, obturator jerk still may be an occasional problem during the resection of the lateral wall located bladder tumors due to the insufficiency of ONB, which may be resulted from the presence of accessory obturator nerve, technical problems, and operator-dependent reasons. It has been hypothesized in the present study that thanks to ONB, bipolar TUR of the lateral-wall located bladder tumors may be safer and more effective than monopolar resection. In order to test this hypothesis, a prospective-randomized study has been designed with the aim of comparison of perioperative outcomes and complications of PlasmaKinetic bipolar and monopolar transurethral resection (TUR) of non-muscle invasive lateral wall-located bladder tumors in patients under obturator nerve block (ONB). As far as is known, the present study is the first one to compare the outcomes and complications of the monopolar and bipolar devices with ONB as a primary end-point.

## PATIENTS AND METHODS

The Institutional Review Board approved the present single-center, prospective and randomized study (IRB no: 031504). Written informed consent form had been obtained from each patient before the study was carried out.

Between March, 2016 and November, 2019, all patients with the suspicion of bladder tumor were preoperatively evaluated by imaging tools and/or cystoscopy and lateral bladder wall-located bladder tumors were selected among them. 80 patients in total, who underwent TURBT of primary, lateral bladder wall-located tumors under ONB, were included in the study.

Anticoagulant/antiaggregant medications, such as acetylsalicylic acid, warfarin, and clopidogrel were stopped 7 days before the procedure and, when necessary, were replaced by low-molecular-weight heparin.

Patients with muscle invasive bladder cancers, variant histologies, neurological disorders affecting the central nervous system, previous surgery and scars in the ONB region, history of allergy to local anesthetic agents, prior incomplete tumor resection as well as recurrent tumors were excluded.

Patients were randomized on odd and even number basis by a computer-based system. Odd numbers for M-TURBT and even numbers for B-TURBT had been implemented until complete recruitment in each group was achieved.

**Figure 1:**
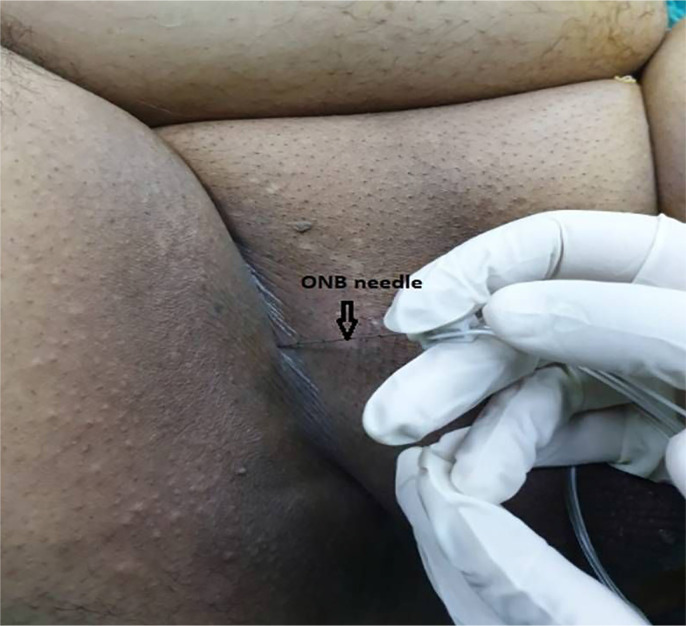
Demonstration of the position and insertion of the obturator nerve block needle (Obturator nerve blockade prior to the transurethral resection of 3 cm tumoral mass on the right lateral bladder wall of a 63- year-old male patient.)

All patients underwent spinal anesthesia combined with ONB by the nerve stimulator. In spinal anesthesia, 10 to 15mg 0.5% hyperbaric bupivacaine was applied by 25G Quincke needle at the L3-L4 or L4-L5 space in the seated position. Afterwards, the patients were immediately placed into the supine position and sensory blockade was checked with a pin-prick test. When the block reached the T10 level, patients were placed into the lithotomy position and ONB was performed unilaterally or bilaterally according to tumor position. A 10mm Teflon-insulated needle (21G Stimuplex A, B. Braun Melsungen AG, Germany) using a nerve stimulator (B. Braun Melsungen AG, Germany) was inserted perpendicularly 2cm inferiorly and 2cm laterally to the pubic tubercle (Figure-1). In nerve stimulator, as in the traditional approach, power current was set to 1.5 to 2mA and current periods were set to 0.1ms. To start with, the needle was inserted through the skin to the inferior ramus of the pubic bone and it was slightly pulled back and redirected anterolaterally contacting the nerve in a depth of 2 to 4cm. After aspiration was negative, when muscle contraction was observed at the adductor muscle groups, 10mL 0.25% levobupivacaine was applied at 0.3 to 0.5mA. Surgery was initiated 10 minutes after injection. The same anesthesia staff performed the ONBs. No additional techniques were utilized in order to prevent adductor muscle contractions during surgery other than ONB.

Perineal skin was cleaned with the antiseptic solution in dorsal lithotomy position. After a routine cysto-urethroscopy, M-TURT was performed with an U-shaped cutting loop, 26Fr continuous flow resectoscope (Karl Storz Endoskope, Tuttlingen, Germany) with a 30-degree telescope, and an electrosurgical generator (Valleylab Force FX, Boulder, CO, USA) with power settings of 120W to cut and 80W to coagulate using mannitol irrigation under spinal anesthesia. B-TURMT was performed with an U-shaped cutting loop, 26Fr continuous flow resectoscope with a 30-degree telescope, and an ESG-400 bipolar generator (Olympus Europe, Hamburg, Germany) with power settings of 200W to cut and 120W to coagulate using saline irrigation under spinal anesthesia.

At the end of the procedure, a 22Fr three-way foley catheter was inserted. Postoperative bladder irrigation proceeded until efflux was clear. In uncomplicated cases in which urine was clear, the catheter was usually removed after 24-48h. The patients who were suspected of having NMIBC underwent early instillation of epirubicin (50mg in 50mL saline solution) in 24h after TURB, according to the guidelines of the European Association of Urology (EAU) (3). All procedures were performed by several experienced surgeons in a tertiary teaching hospital.

The primary outcome of this study was the safety of the procedures, such as obturator jerk, bladder perforation, clot retention, febrile urinary tract infection, and TUR syndrome. Obturator jerk was defined as adductor muscle spasm which disturbed the surgeons during resection. Bladder perforation was defined as subserosal injury when the perivesical fatty tissue was seen and it was defined as complete perforation when drainage tube or surgical repair was required. TUR syndrome was defined as serum sodium level <125mmol/L and as one or more circulatory and/or neurological symptoms. All complications were graded according to Clavien scoring system.

The secondary outcome was the efficacy of both TURBT procedures, including complete tumor resection, sampling of the deep muscle tissue and sampling of the qualified tissues without any thermal damage. Besides, all resections were performed under the supervision of a senior urologist, and completeness and complications of resection were noted intraoperatively.

All resected specimens were evaluated by the same uropathologist who was blinded for allocation. Stage and grade of tumor, presence of muscularis propria, invasion of muscle tissue, and presence of thermal tissue damage were all reported.

Statistical analysis: The data were analyzed using the Statistical Package for Social Sciences (spss 17.0 for Windows, Chicago, IL, USA). The data were expressed as mean±standard deviation, number and percentage according to the type of variables. Normality tests (Shapiro-Wilk test, p >0.05) were performed in order to evaluate the distributions of numerical variables. As long as the distribution of numerical variables was normal, statistical analysis was performed using parametric Student’s t-tests. Mann-Whitney U-tests were used to evaluate numerical variables with a skewed distribution. Categorical variables were analyzed using chi-square or Fisher’s exact tests.

G* Power (3.0.8) statistical software package program was utilized in order to calculate the power of the study. A sample size of 72 achieved 95% power to detect an effect size (W) of 0.50 using a 1 degree of freedom Chi-Square test with a significance level (alpha) of 0.01.

## RESULTS

**Figure 2:**
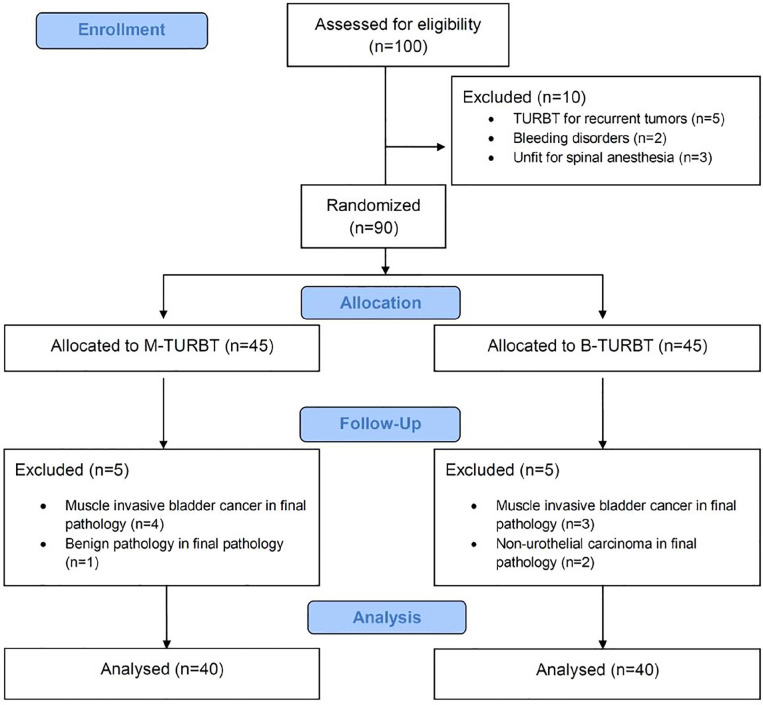
CONSORT (Consolidated Standards for Reporting Trials) flow diagram for patient assessment, allocation, follow-up and analysis.

All patients were equally randomized to M-TURBT and B-TURBT groups. There were 40 patients in each group (Figure-2). Mean ages were 66.88±10.22 and 64.38±9.53 years in M-TURBT and B-TURBT groups, respectively (p=0.261). There were no statistically significant differences in terms of patient demographics (Table-1).

**Table 1:** Demographics and tumor characteristics.

		M-TURBT (N:40)	B-TURBT (N:40)	P
**Sex (n, %)**				0.132*
	Male	34 (85)	38 (95)	
	Female	6 (15)	2 (5)	
Age (years) (mean±SD)		66.88±10.22	64.38±9.53	0.261**
BMI (kg/m^2^) (mean±SD)		27.07±5.08	26.62±4.49	0.673**
**ASA score (n, %)**				0.237*
	1	2 (5)	3 (7.5)	
	2	25 (62.5)	30 (75)	
	3	13 (32.5)	6 (15)	
	4	0 (0)	1 (2.5)	
**Tumor Localization (n, %)**				0.354*
	**Right**	17 (42.5)	16 (40)	
	**Left**	15 (37.5)	20 (50)	
	**Bilateral**	8 (20)	4 (10)	
Tumor size (cm, mean ± SD)		3.75±2.62	4.5±3.13	0.298***
Number of tumor (n, mean ± SD)		1.85±1.17	2.45±2.86	0.992***
**Focality**				0.823*
	Unifocal	20 (50)	21 (52.5)	
	Multifocal	20 (50)	19 (47.5)	
**Stage (n, %)**				0.823*
	Ta	19 (47.5)	20 (50)	
	T1	18 (45)	19 (47.5)	
	T1+CIS	3 (7.5)	1 (2.5)	
**Grade (n, %)**				0.572*
	PUNLP	0 (0)	1 (2.5)	
	Low	30 (75)	28 (70)	
	High	10 (25)	11 (27.5)	

**SD** = Standard deviation; **BMI** = Body mass index; **ASA** = American Society of Anesthesiologists; **CIS** = Carsinoma insitu; **PUNLP** = Papillary urothelial neoplasm of low malignant potential; **TUR** = Transurethral resection

* Chi Square,

** Student-t,

*** Mann Whitney

There were no statistically significant differences between the groups in terms of size and number of tumors, and histopathological properties (stage, grade). There were 17 and 16 right lateral bladder wall-located tumors, 15 and 20 left lateral bladder wall-located and 8 and 4 bilateral lateral bladder wall-located tumors in M-TURBT and B-TURBT groups, respectively (p=0.354) (Table-1).

Obturator jerk was detected in 2 patients (5%) in M-TURBT group while obturator jerk was not observed in B-TURBT group. This difference was not statistically significant (p=0.494). Neither bladder perforation nor TUR syndrome was observed in both groups. All patients in each group underwent complete tumor resection. Cautery artifact was reported in 12.5% and 2.5% of the patients in M-TURBT and B-TURBT groups, respectively. However, the difference was not statistically significant (p=0.201). Muscle tissue sampling rate was comparable between the groups (67.5% in M-TURBT vs. 72.5% in B-TURBT; p=0.626). There were no statistically significant differences regarding mean operation and hospitalization times. However, mean catheterization time was significantly longer in M-TURBT group (4.05±2.91 vs. 3.08±2.19; p=0.018) (Table-2).

**Table 2:** Perioperative Outcomes.

	M-TURBT (n=40)	B-TURBT (n=40)	P
**Obturator jerk (n, %)**	2 (5)	0	0.494*
**Bladder perforation (n, %)**	-	-	
**TUR syndrome (n, %)**	-	-	
**Complete resection (n, %)**	40 (100)	40 (100)	
**Muscle tissue sampling (n, %)**	27 (67.5)	30 (75)	0.459*
**Cautery artifact (n, %)**	5 (12.5)	1 (2.5)	0.201*
**Hb decrease (g/dL) (mean+SD)**	-0.76±0.75	-0.60±0.79	0.347***
**Na decrease (mmol/L) (mean+SD)**	-1.25±3.40	-0.33±2.65	0.428***
**Operation time (min) (mean±SD)**	34.13±23.53	35.50±15.80	0.208***
**Catheterization time (day) (mean± SD)**	4.05±2.91	3.08±2.19	0.018***
**Hospitalization time (day) (mean± SD)**	2.15±1.31	2.20±1.09	0.598***

**SD** = Standard deviation; **TUR** = Transurethral resection; **Hb** = Hemoglobin; **Na** = Sodium, min: minute

* Chi Square,

*** Mann Whitney U

The number of uncomplicated cases in M-TURBT and B-TURBT groups was 20 (50%) and 27 (67.5%), respectively (p: 0.112). All complications were classified according to Clavien scoring system. The majority of the complications were low-grade and the differences regarding low-grade complications between the groups were not statistically significant (p=0.235 for Clavien grade-1, p=0.805 for Clavien grade-2). Severe complications (≥ Clavien grade-3) were observed in 3 patients in M-TURBT group while no severe complication was noted in B-TURBT group (p=0.077). Of these severe cases, 1 patient required percutaneous nephrostomy insertion due to severe hydronephrosis and 2 patients required endoscopic cauterization because of active hematuria (Table-3).

## DISCUSSION

Transurethral resection of bladder tumor remains the gold standard for proper diagnosis, correct staging, and initial treatment of NMIBCs. However, residual tumor after initial TURBT is related to a higher risk of disease recurrence (3). Therefore, complete and optimal TURBT is crucial to achieve successful oncological outcomes.

During the resection of the bladder tumors, it is common to see the occurrence of obturator reflex. Obturator jerk increases the risk of some complications, such as incomplete resection, bladder perforation, the inability to give intravesical chemotherapeutic agents, extravesical dissemination of the tumor, and vascular injuries (12). Most urologists are concerned about some possible complications that may occur during resection of lateral wall-located bladder tumors when obturator nerve is close to lateral wall in its intrapelvic course (19). Depending on the resectoscope type, anesthesia technique, and ONB procedure, the previous studies reported that obturator jerk was detected in 2.1-82.6% of the patients during the TUR of lateral and inferolateral bladder tumors (18, 20, 21). Furthermore, at least half of the bladder tumors are located on the lateral wall (13). In a study of Liem et al. which involves 716 patients, the authors demonstrated that 76.6% of the unifocal tumors and 84.6% of the multifocal tumors are located in the lateral wall of the bladder (5).

Additional measures should be taken during the resection of the tumors on the lateral wall. Different strategies, such as partial filling of the bladder during resection, reducing the intensity of the current of the resectoscope, resecting the tumor on thinner slices, using bipolar or laser resectoscopes, and using general anesthesia with muscle relaxants are adopted to avoid complications during surgery (8, 22).

In order to avoid the obturator jerk and related complications, one of the most common measures is blockade of the obturator nerve prior to TURBT. In the prospective-randomized study which was published in 2015 by the authors of the present study, the efficacy of ONB on adductor muscle spasm and related outcomes during monopolar TURBT of lateral wall-located bladder tumors were investigated (18). It was reported that severe obturator jerk which was defined as severe enough to disturb the surgeon’s resection was detected in 17.1% of patients under spinal anesthesia and in 2.8% patients under combined spinal anesthesia and ONB. Despite the favorable complication rates, such as complete bladder perforation (0 vs. 5.7%), and incomplete resection (2.9% vs. 22.9%) in ONB group, mean operation time was comparable.

Tekgul et al. compared the recurrence rates of patients with lateral wall-located bladder tumors having undergone TURBT with or without ONB and they reported that ONB performed in addition to spinal anesthesia in TURBT procedures can prolong time to recurrence and increase the chance to lengthen the disease-free survival in low-risk superficial bladder tumors (23).

Furthermore, Erbay et al. reported that spinal anesthesia, combined with ONB, during TURBT prevents obturator reflex and facilitates complete resection including detrusor muscle tissue, independent from the size and number of tumors, and thus reduces the recurrence of the disease (20). Despite the ONB, obturator jerk was still observed in some cases. This can be explained by the presence of the accessory obturator nerve. About 10% to 30% of the population has accessory obturator nerve (24, 25). It lies parallel to the main obturator nerve along the medial side of the psoas muscle passing ventral to the superior pubic ramus behind the femoral vein and joins the anterior part of the main obturator nerve (25). It is debatable whether stimulation of accessory obturator nerve can occur during TURBT. Failure of blockade can also be related to the operator error. It may be difficult for an inexperienced surgeon to find the location of obturator nerve; therefore, the blockade cannot be done successfully.

Another alternative approach, which could be adopted in order to decrease the obturator reflex related to complications, may be using bipolar PlasmaKinetic resectoscopes (5, 26). In a randomized prospective study in 2016 published by the authors of the present study, perioperative outcomes and complications of PlasmaKinetic bipolar and monopolar TURBT in patients with NMIBC regardless the tumor location were compared (26). As a result, the incidence of obturator reflex was 21.5% in M-TURBT group and 4.6% in B-TURBT group (p=0.013). Moreover, severe degree obturator jerk was observed in 5 (7.7%) vs. 2 (3.1%) patients in M-TURBT and B-TURBT groups, respectively. Ozer et al. compared monopolar and bipolar techniques in terms of bladder injury due to obturator reflex in patients who underwent TUR for NMIBC, and they noted more obturator reflex and reflex-related bladder perforation in bipolar group (27). Recently, Gramann et al. published the results of a randomized-prospective study which was carried out with 44 patients and investigated the superiority of B-TURBT to M-TURBT on newly diagnosed or recurrent lateral bladder wall tumors (21). All operations were performed under laryngeal mask anesthesia without ONB and without drug-induced relaxation. Complete tumor resection rates without clinically significant obturator jerk were reported as 61.9% vs. 82.6% in M-TURBT and B-TURBT groups, respectively (p=0.18). Additionally, the authors supported ONB when using spinal anesthesia and drug-induced relaxation during TURBT on the lateral bladder wall.

Abovementioned studies demonstrated that the use of bipolar resectoscopes is not enough to avoid the obturator reflex and related-complications. Different tumor locations and anesthesia techniques may have significant effects on the occurrence of obturator jerk; therefore, prior to TUR procedure, ONB can be recommended for bladder tumors located on the lateral wall.

In the light of the abovementioned cases, in order to overcome obstacles that could be encountered during ONB and insufficiencies of monopolar and bipolar resectoscopes, it was aimed to design a prospective-randomized study to compare the safety and efficiency of M-TURBT and B-TURBT of the lateral wall-located bladder tumors under ONB. As far as it is known, the present study is the first one to compare the outcomes and complications of different resectoscope types under ONB. In the present study, obturator jerk was detected in 2 patients (5%) in M-TURBT group while no obturator jerk was observed in B-TURBT group (p=0.494). In these two patients, resectoscope was changed from monopolar system to bipolar and resection was completed without any obturator jerk. Bladder perforation was not observed in both groups. All patients in each group underwent complete tumor resection. Accordingly, muscle tissue sampling rate was comparable between the groups. All these findings demonstrate that under the ONB, both M-TURBT and B-TURBT can be performed effectively.

In their previous study, the authors of the present study compared the short-term outcomes and complications of monopolar and bipolar transurethral resection of bladder tumors in patients with coronary artery disease and they reported that neither was superior to each other regarding bladder perforation, febrile urinary tract infection or hematuria (28). In the present study, in accordance with the previous one, overall complication rates were similar between both arms. The majority of the complications were low-grade, and the low-grade complications differences between the groups were not statistically significant. Severe complications (≥ Clavien grade-3) were observed in 3 patients in M-TURBT group, while no severe complication was noted in B-TURBT group (p=0.077). Of these cases, 1 patient required percutaneous nephrostomy insertion due to severe hydronephrosis and 2 patients required endoscopic cauterization owing to active hematuria.

Although mean operation and hospitalization times were similar between the two arms, mean catheterization time was significantly longer in M-TURBT group (4.05±2.91 vs. 3.08±2.19; p=0.018). Moreover, despite not having any statistically significance, cautery artifact was also more common in M-TURBT group. When these two situations are considered together, it can be concluded that surgical experience may be important in M-TURBT procedure. However, as there is no data on surgical experience, it is not possible to provide an accurate explication about it. These results demonstrate that under ONB either M-TURBT or B-TURBT can be performed safely. However, hemorrhagic complications such as cauterization-requiring hematuria must be considered in patients undergoing M-TURBT. Thus, more attention should be paid for hemostasis during the procedure.

The present study has some limitations. Firstly, the sample size is relatively small. However, the power analysis demonstrated that a sample size of 72 patients was sufficient to get a 95% power. This can be explained by the rigid criterion of inclusion. Namely, only the lateral wall-located NMIBCs were investigated, and otherwise located or muscle invasive bladder cancer (MIBCs) were excluded. Secondly, ONB procedures and operations were not performed by the same operators. Although the procedures were performed by several experienced specialists, it was not shown in the results. Lastly, as the oncological results were not the primary or secondary outcomes of the study, recurrence and progression free survivals were not assessed.

## CONCLUSION

At least half of the bladder cancers are located on the lateral walls of the bladder. In the treatment of lateral-wall located NMIBCs, either M-TURBT or B-TURBT can be safely and effectively performed under the combination of spinal anesthesia and ONB in order to avoid the obturator jerk and related complications. However, it should be taken into account by especially new-experienced surgeons that postoperative hemorrhagic complications may arise in patients undergoing M-TURBT, and thus more attention should be paid for hemostasis during the procedure.
